# HRS/EHRA/APHRS/LAHRS/ACC/AHA Worldwide Practice Update for Telehealth and Arrhythmia Monitoring During and After a Pandemic

**DOI:** 10.1161/CIRCEP.120.009007

**Published:** 2020-07-21

**Authors:** Niraj Varma, Nassir F. Marrouche, Luis Aguinaga, Christine M. Albert, Elena Arbelo, Jong-Il Choi, Mina K. Chung, Giulio Conte, Lilas Dagher, Laurence M. Epstein, Hamid Ghanbari, Janet K. Han, Hein Heidbuchel, He Huang, Dhanunjaya R. Lakkireddy, Tachapong Ngarmukos, Andrea M. Russo, Eduardo B. Saad, Luis C. Saenz Morales, Kristin E. Sandau, Arun Raghav M. Sridhar, Eric C. Stecker, Paul D. Varosy

**Affiliations:** 1 Cleveland Clinic, OH (N.V., M.K.C.).; 2 Tulane University School of Medicine, New Orleans, LA (N.F.M., L.D.).; 3 Centro Privado de Cardiología, Tucuman, Argentina (L.A.).; 4 Cedars-Sinai Medical Center, Los Angeles, CA (C.M.A.).; 5 Arrhythmia Section, Cardiology Department, Hospital Clínic, Universitat de Barcelona, Spain. Institut d’Investigacións Biomèdiques August Pi i Sunyer (IDIBAPS), Barcelona, Spain (E.A.).; 6 Centro de Investigación Biomédica en Red de Enfermedades Cardiovasculares (CIBERCV), Madrid, Spain (E.A.).; 7 Korea University Medical Center, Seoul, Republic of Korea (J.-I.C.).; 8 Cardiocentro, Lugano, Switzerland (G.C.).; 9 Northwell Health, North Shore University Hospital, Manhasset, New York (L.M.E.).; 10 University of Michigan, Ann Arbor (H.G.).; 11 VA Greater Los Angeles Healthcare System and David Geffen School of Medicine at the University of California (J.K.H.).; 12 Antwerp University and University Hospital, Belgium (H. Heidbuchel).; 13 Renmin Hospital of Wuhan University, China (H. Huang).; 14 Kansas City Heart Rhythm Institute and Research Foundation, Overland Park (D.R.K.).; 15 Faculty of Medicine Ramathibodi Hospital, Mahidol University, Bangkok, Thailand (T.N.).; 16 Cooper Medical School of Rowan University, Camden, NJ (A.M.R.).; 17 Hospital Pró-Cardíaco, Rio de Janeiro, Brazil (E.B.S.).; 18 CardioInfantil Foundation, Cardiac Institute, Bogota, Colombia (L.C.S.M.).; 19 Bethel University, St. Paul, MN (K.E.S.).; 20 University of Washington, Seattle (A.R.M.S.).; 21 Oregon Health & Science University, Portland, OR (E.C.S.).; 22 VA Eastern Colorado Health Care System and University of Colorado, Aurora (P.D.V.).

**Keywords:** COVID-19, pandemic, QT interval, remote monitoring, telemedicine

Coronavirus disease 2019 (COVID-19), caused by the severe acute respiratory syndrome coronavirus 2, started in the city of Wuhan late in 2019. Within a few months, the disease spread toward all parts of the world and was declared a pandemic on March 11, 2020. The current healthcare dilemma worldwide is how to sustain the capacity for quality services not only for those suffering from COVID-19 but also for non-COVID-19 patients, all while protecting physicians, nurses, and other allied healthcare workers.

The pandemic poses challenges to electrophysiologists at several levels. Hospitalized patients who are COVID-19-positive may have preexisting arrhythmias, develop new arrhythmias, or be placed at increased arrhythmic risk from therapies for COVID-19. Cardiac arrhythmia incidence in hospitalized patients has been documented in a few published studies, with reported rates of 7.9%^1^ and 16.7%^2^ in hospitals in New York City and Wuhan, respectively, and up to 44%^2^ in patients requiring intensive care. Life-threatening arrhythmias (ventricular tachycardia/ventricular fibrillation) can occur in up to 6% of hospitalized patients with COVID-19 infection.^3^ There have also been several case reports of atrioventricular block in hospitalized patients, which is otherwise rarely described during viral illness.^4,5^ Although the residual left ventricular dysfunction and arrhythmic risk are currently unknown, preliminary pathophysiological,^6^ histological,^7^ and imaging^8^ data suggest that severe acute respiratory syndrome coronavirus 2 infection holds the potential to induce durable myocardial changes predisposing to arrhythmias or heart failure.

Electrocardiographic monitoring and inpatient monitoring services may become necessary but face the potential hurdles of limited telemetry wards, contamination of equipment and infection of healthcare personnel, and shortage of personal protective equipment.^9,10^ In parallel, there is a continued responsibility to maintain care of patients who are COVID-19-negative with arrhythmias. These pressures have led to inventive utilization and adaptation of existing telemedicine technologies as alternative options.

This document discusses how digital health may facilitate electrophysiology practice for patients with arrhythmia, whether hospitalized for COVID-19 or not. The representation of authors from some of the most severely affected countries, such as China, Spain, Italy, and the United States, is a tribute from our worldwide community to those colleagues who have worked on the front lines of the pandemic.

## Monitoring Strategies During a Pandemic: Here to Stay

In light of the current pandemic, monitoring strategies should focus on selecting high-risk patients in need of close surveillance and using alternative remote recording devices to preserve personal protective equipment and protect healthcare workers from potential contagious harm.

### Inpatient

For inpatient monitoring, telemetry is reasonable when there is concern for clinical deterioration (as may be indicated by acute illness, vital signs, or sinus tachycardia), or in patients with cardiovascular risk factors and/or receiving essential QT-prolonging medications. Telemetry is generally not necessary for persons under investigation without concern for arrhythmias or clinical deterioration and in those not receiving QT-prolonging drug therapy. In situations in which a hospital’s existing telemetry capacity has been exceeded by patient numbers or when conventional telemetry monitoring is not feasible, such as off-site or nontraditional hospital units, mobile devices may be used, for example, mobile cardiac outpatient telemetry as an adjunctive approach to support inpatient care.^11–15^ The majority of mobile cardiac outpatient telemetry devices can provide continuous arrhythmia monitoring using a single-lead ECG and allow for real-time and offline analysis of long-term ECG data. Telemetry can be extended using patch monitoring.^16,17^ Smartphone ECG monitors are wireless and have also been utilized during the current pandemic. Information is limited, however, on how parameters such as QTc measured on a single- (or limited number) lead ECG can reliably substitute for 12-lead ECG information.^18,19^ In one study, QT was underestimated by smartphone single-lead ECG.^20^

### Outpatient

The principles of remote patient management, crossing geographic, social, and cultural barriers, can be extended to outpatient care and are important to maintain continuity of care for non-COVID-19 patients.^21–23^ Virtual clinics move far beyond simple telephone contacts by integrating information from photos, video, mobile heart rhythm, and mobile health devices recording ECG, and remote cardiovascular implantable electronic device (CIED) interrogations.^24^ A variety of platforms have been developed and used specifically to provide telehealth to patients via video teleconferencing^25,26^ (Table 1). Most healthcare centers have expanded use of telemedicine, with some reporting 100% transformation of in-person clinic visits to telemedicine-based visits to maintain care for non-COVID-19 patients, thus obviating their need to come to the hospital or clinic. This supplements social distancing measures and reduces the risk of transmission, especially for the older and more vulnerable populations. It also becomes a measure to control intake into emergency rooms and outpatient facilities and to permit rapid access when necessary to subspecialists.

**Table 1.**
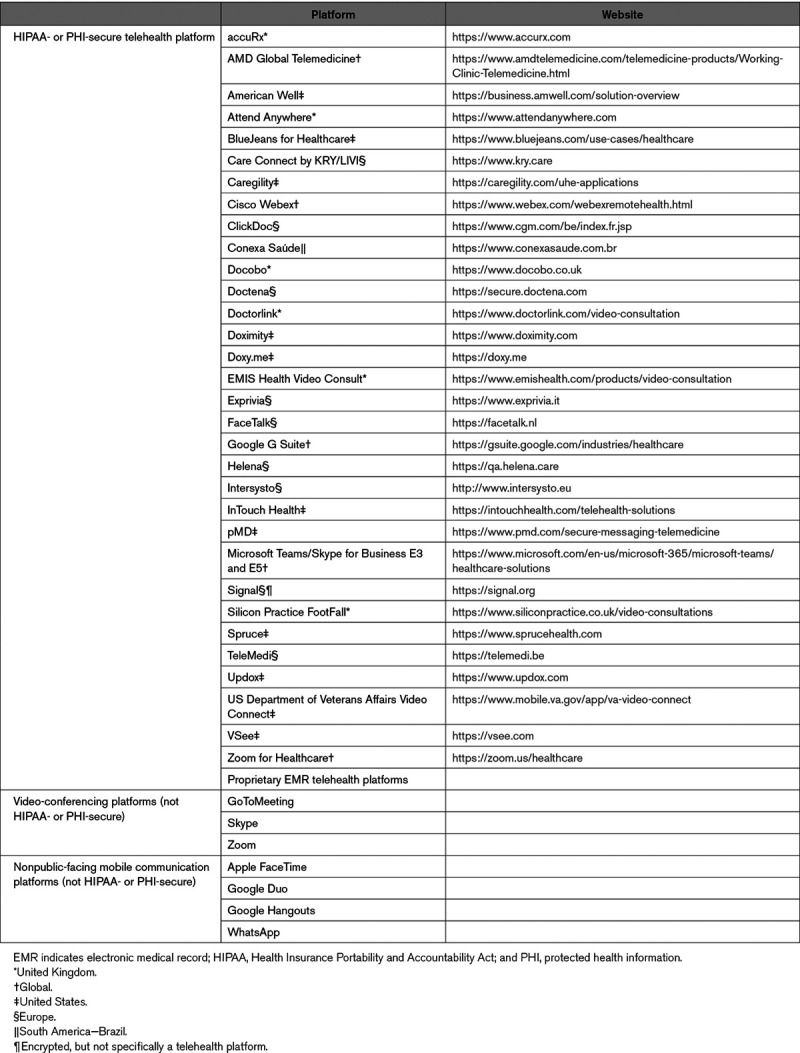
Examples of Commonly Used Platforms for Telehealth^25–27^

Electrophysiology is well placed for virtual consultations. All preobtained data, including ECGs, ambulatory ECG monitoring, cardiac imaging, and coronary angiography can be adequately reviewed electronically. Digital tools such as direct-to-consumer mobile ECG (Table 2) and wireless blood pressure devices can be used to further complement the telehealth visit without in-person contact. CIED, wearable/mobile health, and clinical data can be integrated into clinician workflow.

**Table 2.**
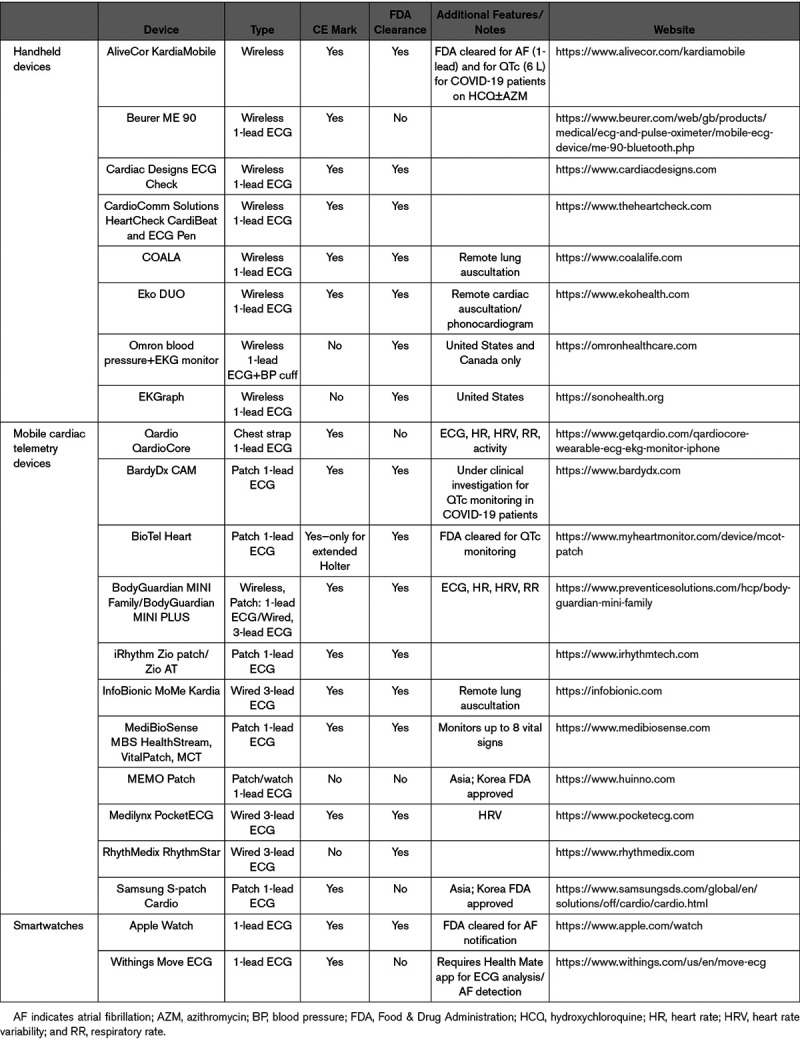
Examples of Remote ECG and Heart Rate Monitoring Devices

Additional diagnostic information might be obtained without in-person contact using home enrollment of prescribed ambulatory rhythm monitors. Patch monitors can be mailed to patient homes and easily self-affixed, unlike Holter monitors with cables and electrodes requiring placement by healthcare workers. In some cases, new or follow-up telehealth visits will require an adjunctive in-person visit to perform a 12-lead ECG, ECG stress test, echocardiogram, or other diagnostic procedures. Occasionally, conventional clinic visits are required to accurately assess the impact of comorbidities or frailty on procedural risk, or to allow comfortable discussion with multiple family members when planning procedures with high risk. Telephone-only visits (ie, without video) may allow for a broader reach because of ease and ubiquitous accessibility as a communication strategy for immediate access for urgent matters.

There are many barriers to implementation, such as inadequate reimbursement, licensing/regulatory and privacy issues, lack of infrastructure, resistance to change, lack of access/poor internet coverage, restricted financial resources, and limited technical skills (eg, in the elderly patient population). Some telehealth and remote ECG monitoring technologies may be simply unaffordable and/or unavailable, leading to different levels of uptake within communities and across the globe. All stakeholders should collaborate to address these challenges and promote the safe and effective use of digital health during the current pandemic. In recent months, regulations have been eased to permit consults with new patients, issuing prescriptions, and obtaining consents. In that sense, the COVID-19 pandemic may serve as an opportunity to evolve current technologies into indispensable tools for our future cardiological practice.

## Therapy for COVID-19 and Potential Electrical Effects

No specific cure exists for COVID-19.^28–30^ Potential COVID-19 therapies, especially hydroxychloroquine and azithromycin, are being investigated in ongoing trials but also have been used off label in many parts of the world. These may exert QT-prolonging effects^31^ (Table 3) and, since recent observational data have questioned their efficacy, require a careful risk-benefit adjudication.^32^ Combination therapy (eg, hydroxychloroquine and azithromycin) may have synergistic effects on QT prolongation.^33,34^ In a retrospective cohort study of 1438 patients with COVID-19 hospitalized in metropolitan New York (ie, a disease epicenter), cardiac arrest was more frequent in patients who received hydroxychloroquine with azithromycin compared with patients who received neither drug.^35^ The adjusted hazard ratio for in-hospital mortality for treatment with hydroxychloroquine alone was 1.08, for azithromycin alone was 0.56, and for combined hydroxychloroquine and azithromycin was 1.35. However, none of these hazard ratios were statistically significant. The observational design of this study may limit interpretation of these findings. In the absence of clear efficacy data, treatment options should be individualized taking into account their proarrhythmic potential for torsade de pointes, which may be enhanced by concomitant administration of other QT-prolonging drugs (eg, antiarrhythmics, psychotropics, etc).

**Table 3.**
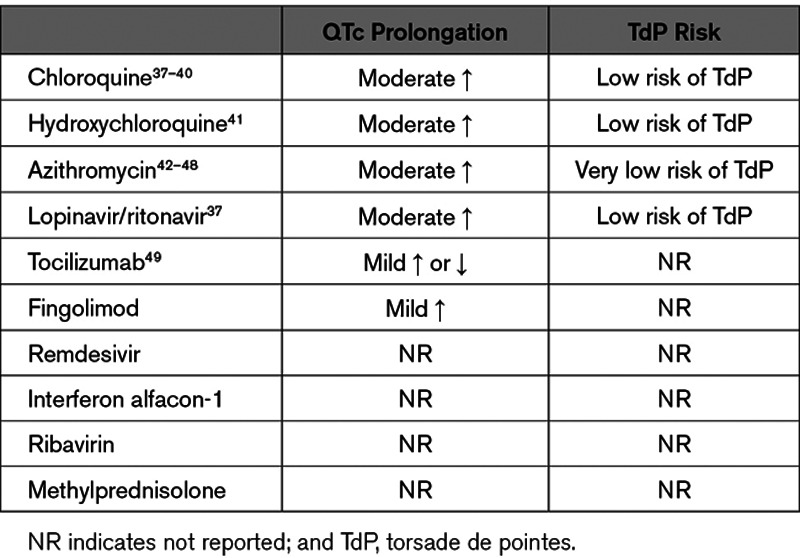
Effect on QTc and Proarrhythmia of Experimental Pharmacological Therapies for COVID-19^36^

In COVID-19 patients receiving prior antiarrhythmic therapy, there should be a thorough consideration of risk versus benefit before initiating any QT-prolonging COVID-19 therapies, especially considering their unproven value. For instance, although some recent observational studies highlight adverse effects of hydroxychloroquine in treating this infectious disease, its use is likely to persist outside of randomized trials because of its affordability and global availability compared with, for example, remdesivir.^35^ If one of these drugs is judged to be critical, monitoring should be initiated. If life-threatening arrhythmias (ventricular tachycardia/ventricular fibrillation) occur, the benefit of antiarrhythmic drugs, notably amiodarone, outweighs the potential harm of hydroxychloroquine or other QT-prolonging drugs targeting COVID-19, and in these cases antiarrhythmic drugs should be prioritized and used as deemed necessary. Most importantly, all modifiable predisposing factors for QTc prolongation (electrolyte disturbances, drug-to-drug interaction) that may enhance arrhythmia susceptibility should be corrected, and the small subset of individuals with an underlying genetic predisposition such as congenital long QT syndrome (in whom QTc-prolonging medications are contraindicated) should be identified. Additionally, caution must be exercised in case of subclinical or manifest myocarditis that may increase the vulnerability to proarrhythmias associated with QT-prolonging drugs.

If drugs that exert a QT-prolonging effect are to be initiated in an inpatient setting, a baseline 12-lead ECG should be acquired. Following review of the QTc, patients can be stratified into low-risk group (QTc of <500 ms or <550 ms in the setting of wide baseline QRS) or high-risk group (baseline QTc of ≥500 ms or ≥550 ms in the setting of wide baseline QRS, or patients who are started on combination therapies), guiding selection of telemetered versus nonmonitored beds.^50^ Low-risk patients treated with QT-prolonging agents may be monitored using MCT (or another available wearable) with twice-a-day transmission of QTc measurements and any urgent alerts. High-risk patients would require more continuous monitoring and follow-up QTc measurements using telemetry preferably (but if unavailable, other remote monitoring devices). A second QTc assessment via telemetry or other remote devices after 2 doses may be helpful in identifying QTc reactors—patients who have an exaggerated response to QT-prolonging agents. An increase in QTc by ≥60 ms or to QTc ≥500 ms on any follow-up QT assessment is considered significant and should prompt a reassessment of risks versus benefits of continuing the drug.

In the outpatient setting, a recent statement from the US Food & Drug Administration (FDA) “cautions against use of hydroxychloroquine or chloroquine for COVID-19 outside of the hospital setting or a clinical trial due to risk of heart rhythm problems.” (This does not affect FDA-approved uses for malaria, lupus, and rheumatoid arthritis.)^51^ Exceptions to this practice are acknowledged to occur in some regions, as these drugs have been used outside the United States without regulatory warnings. Under these conditions, or when these drugs are maintained after hospital discharge, consumer mobile ECG devices capable of generating QTc measurements may be used. If the QTc increases significantly, physicians can consider a change or discontinuation of medication via the phone or virtual medical services.

### Electrocardiographic Monitoring During Clinical Trials

Several double- and multi-arm blind randomized controlled trials are underway worldwide for COVID-19 outpatients utilizing different medications that may prolong the QT interval.^52–56^ These drugs are being tested either alone or in various combinations and are being compared with one another, with differential dosing regimens and/or placebo. These drugs are also being tested for postexposure prophylaxis in high-risk groups.

Mobile health using smartphone-based portable ECG devices as QTc monitoring tools is an innovative and economical solution to conduct monitoring in outpatient trials. For instance, in one trial evaluating hydroxychloroquine and azithromycin (hydroxychloroquine alone and hydroxychloroquine/azithromycin combination) against a placebo, participants receive remote training to acquire a 6-lead ECG at baseline and then at specified follow-up intervals through the trial period (Figure). These ECGs are transmitted to a remote QTc monitoring site, where the QTc is assessed and monitored over the treatment period.

**Figure.**
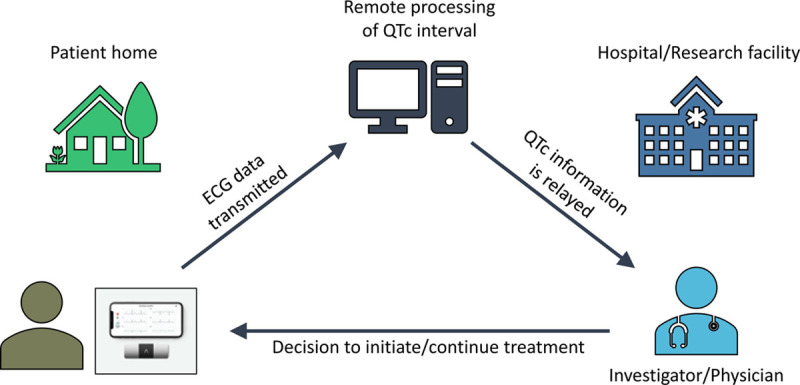
**ECG acquisition and transmission using a smartphone-based portable ECG monitor.**

## The Future: Digital Medicine Catalyzed by the Pandemic

The COVID-19 public health emergency has forced changes to traditional norms of health care access and delivery across all continents.^10^ It has accelerated adoption of telemedicine and all aspects of digital health, regarded as a positive development. Today’s new reality will likely define medicine going forward. Many monitoring and diagnostic testing aspects of both inpatient and outpatient care will be increasingly served by digital medicine tools.

The need for contactless monitoring for inpatients triaged to intensive care unit, telemetry, or nonconventional environments, as well as for outpatients needing continued management, has triggered novel implementation of digital health monitoring tools. Some centers have created algorithms based on predictive analytics of electronic medical record data. Centralized monitoring or mobile continuous monitoring has improved patient outcomes, reduced manpower needs, and is being utilized more commonly.^57^ The use of wearables such as watches, smartphones, and smart beds (with elimination of cables and skin electrodes) for in-hospital telemetry is a novel approach. This type of wireless monitoring may be continued after discharge, permitting prolonged surveillance of rhythm and other physiological parameters.^12^ Bracelet technologies may transmit multiple parameters (eg, heart rate, sleep, oxygen desaturation index, blood pressure) via a smartphone link to centralized hubs. These technologies provide a solution for intensive monitoring extending beyond the hospital environment.

Outpatient management has been revolutionized since the start of the pandemic. Social distancing measures and restricted clinic access have driven the rapid adoption of telehealth mechanisms to continue management of non-COVID-19 patients. Virtual visits that have been used for decades to reach isolated communities,^58^ but less commonly utilized in advanced health systems, have now become the mainstay of ambulatory care across all subspecialties. The initial experience appears to have been positive for both patient and caregiver. Heart rhythm professionals are fortunate to have a choice of wireless technologies to relay monitored information to maintain connection.^12^ Wearable and smartphone-based devices allow convenient real-time monitoring for arrhythmias on a long-term basis because of the comfort associated with their small size and ease of use while reducing patient and healthcare worker exposure. Remote CIED monitoring has existed for decades.^24^ It is strongly endorsed by professional societies, but in practice only a fraction of its diagnostic and therapeutic capabilities has been utilized—until now.^59^ Since the start of the pandemic, utilization of wireless communication with CIEDs has grown exponentially, permanently altering the future of device follow-up. Patient outcomes may be improved with intensive device-based monitoring compared with traditional in-clinic evaluations at regular intervals.^60^ Recent data indicate that in-person CIED evaluation can be extended safely to at least biennially when daily digital connectivity is maintained.^61^ Remote monitoring has the potential advantage of detecting and alerting caregivers (and in the future—patients directly) about important parameter changes, enabling earlier patient hospitalization, even during a presymptomatic phase.^62^

Connectivity permits longitudinal follow-up, with advantages ranging from individual disease management to assessment of penetration of recommended therapies into communities.^60,63^ The ability for CIED remote monitoring data to be streamed to or accessed by multiple providers can facilitate communication and cooperative treatment and should be encouraged. This will require approval by patients, regulators, and manufacturers. Lessons learned from implantable devices can be applied widely in telemedicine. Regulatory bodies have been responsive, for example, approving smartphone-based QT interval measurement and telehealth services across state lines in the United States. The pandemic experience should serve as an impetus to expedite the resolution of persistent challenges, such as validation of digital technologies, infrastructure for data management (and mechanism for relay to patient and caregiver), interoperability with electronic medical record, application of predictive analytics, cybersecurity (and with it the capability for limited forms of remote CIED programming), and reimbursement.^64–66^

In summary, the crisis precipitated by the pandemic has catalyzed the adoption of remote patient management across many specialties and for heart rhythm professionals, in particular. This practice is here to stay—it will persist even if other less arrhythmogenic treatment strategies evolve for COVID-19 and after the pandemic has passed. This is an opportunity to embed and grow remote services in everyday medical practice worldwide.

## Disclosures

None.
